# *Mycobacterium Tuberculosis* and Nontuberculous Mycobacteria Isolates from Presumptive Pulmonary Tuberculosis Patients Attending A Tertiary Hospital in Addis Ababa, Ethiopia

**DOI:** 10.4314/ejhs.v31i1.3

**Published:** 2021-01

**Authors:** Daniel Kahase, Kassu Desta, Zelalem Yaregal, Bazezew Yenew, Getu Driba, Hilina Molalign, Absra Solomon, Fitsum Bekele

**Affiliations:** 1 Department of Medical Laboratory Sciences, College of Medicine and Health Sciences, Wolkite University, South Nation Nationality and peoples, Ethiopia; 2 Department of Medical Laboratory Sciences, College of Health Sciences, Addis Ababa University, Addis Ababa, Ethiopia; 3 National Tuberculosis Laboratory, Ethiopian Public Health Institutes, Addis Ababa, Ethiopia

**Keywords:** Nontuberculous mycobacteria, Multi drug resistant tuberculosis, Mycobacterium tuberculosis complex

## Abstract

**Background:**

Mycobacterial infections are known to cause a public health problem globally. The burden of pulmonary disease from nontuberculous mycobacteria is reportedly on the rise in different parts of the world despite the fact that there is limited data about the disease in sub-Saharan Africa including Ethiopia. Hence, we aimed to assess the magnitude of M. tuberculosis and nontuberculous mycobacteria (NTM) among presumptive pulmonary tuberculosis patients attending St. Paul's hospital Medical College, Addis Ababa, Ethiopia.

**Methods:**

A cross-sectional study was conducted from June to September 20/2016. Morning sputum specimens were collected, processed and cultured in Lowenstein Jensen medium and BACTEC MGIT 960 media. The nontuberculous mycobacteria were further confirmed and characterized by Genotype CM/AS assays. The socio-demographic, clinical and chest x-ray data were collected using a structured questionnaire. The data was analyzed using SPSS version 20.

**Results:**

Out of 275 presumptive tuberculosis patients enrolled in the study, 29(10.5%) were culture positive for Mycobacteria. Of these, 3(10.3%) were found to be NTM and 26(89.6%) were Mycobacterium tuberculosis complex. Of the NTM, two were unidentified and one typed as M.peregrinum. There was no co-isolation of Mycobacterium tuberculosis complex and nontuberculous mycobacteria. Overall, 6(23.1%) Mycobacterium tuberculosis complex isolates were resistant to at least one anti-tuberculosis drug. Of these, two were multidrug resistant tuberculosis cases (7.7%) detected from previously treated patients.

**Conclusion:**

Relatively low magnitude of Mycobacterium tuberculosis complex and nontuberculous mycobacteria isolates were seen in the study area. Therefore, further study using a large sample size is needed to be done to consider nontuberculous mycobacteria infection as a differential diagnosis in presumptive pulmonary tuberculosis patients.

## Introduction

The genus *Mycobacteria* includes aerobic and non-motile organisms. They have a lipid rich cell wall which is thicker than most other bacteria (1). Both *Mycobacterium tuberculosis* and nontuberculous mycobacteria (NTM) classified in this genus are acid fast bacilli indistinguishably and cause chronic lung infections (2,3). They also share clinical manifestation, features of hardiness, hydrophobicity, aerosolization and intracellular pathogenicity (4,5). However, person-to-person transmission has not been convincingly reported in NTM pulmonary disease (6).

Non-tuberculous mycobacteria encompass all *Mycobacterium* species that are not members of the *Mycobacterium tuberculosis* complex and *Mycobacterium leprae*. They are free-living organisms found ubiquitously in the environment mainly in soil and water. More than 125 species of NTM are identified, approximately 60 of which are suspected or known to be pathogenic (7,8). NTM can cause chronic pulmonary disease, disseminated infection, lymphadenitis, and skin and soft tissue infection in both immunocompetent and immunocompromised individuals (7, 8, 9).

Globally, over the past three decades prevalence of pulmonary NTM disease has increased dramatically (10). These could be partially due to factors like the development of microbiological isolation and identification techniques, pathogen, host and host parasite interaction factors (11). Increasing numbers of immune compromised patients, including those with Human Immune Deficiency Virus infection and hematological disorders are also implicated in increment of pulmonary disease due to NTM (8). Studies done in Canada and US indicate that pulmonary NTM (PNTM) disease is far more common than TB and causes higher morbidity than TB especially in elderly individuals (12,13).

Ethiopia is one of the thirty high burden tuberculosis countries in the world (14). According to the 2019 World Health Organization's global TB report, there were an estimated 165,000 incidence cases of tuberculosis (TB,) and the estimated TB cases with MDR/RR-TB in new and previously treated cases were 0.71% and 16% respectively (14).

A lot of studies were conducted regarding the burden of *Mycobacterium tuberculosis* and their drug resistance profile in Ethiopia (15,16,17,18,19,20,21). However, in Ethiopia, very few studies report existence of nontuberculous mycobacteria without species identification (15,20). In addition, drug resistance profile and burden of the tuberculosis disease also need to be updated for effective management of tuberculosis patients. Accordingly, this study focused on assessing the magnitude of *Mycobacterium tuberculosis* and nontuberculous mycobacteria isolates and also determining the drug resistance profile of *Mycobacterium tuberculosis* isolates from presumptive pulmonary tuberculosis patients attending St. Paul's Hospital Millennium Medical College Addis Ababa, Ethiopia.

## Materials and Methods

**Study design and Study population**: A cross sectional study design was done from June to September 20/2016. Presumptive pulmonary tuberculosis patients who were attending St. Paul's Hospital Millennium Medical College were enrolled to the study. It is the second largest public hospital in the nation which serves more than 110,000 people annually.

**Sample size determination and sampling technique**: The minimum required sample size of the study was determined using the formula for a single population proportion. By considering prevalence of pulmonary tuberculosis as 21.3% from previous study done in Addis Ababa, Ethiopia (21), 95% confidence level and marginal error of 5%, the final sample size was 258. However, a total of 275 patients with presumptive pulmonary tuberculosis were consecutively included in this study.

**Data collection**: After informed consent, sociodemographic, health and behavioral factors, clinical history and chest x-ray data were collected from each participants using a structured questionnaire. In addition, about 3–5 ml of morning sputum samples were collected using sterile, disposable, single-use, screw-capped conical centrifuge tubes. It was immediately transported to the national tuberculosis laboratory of Ethiopian public health institute for laboratory processing.

**Sample processing and smear microscopy**: Sputum samples were mixed with an equal volume of N-acetyl-L-cysteine-sodium hydroxide (NALC-NAOH) solution and vortexed for less than 20 seconds and kept for 15 minutes at 20–25 °C for the decontamination. Phosphate Buffered saline (PBS) was filled to a 50 ml mark on the falcon tube and vortexed. It was centrifuged at 3000 g for 15 minutes and the supernatant was discarded. A portion of the sediments was used for culture and the other portion for Ziehl-Neelsen staining after resuspended in 1 ml PBS (22).

Sputum smears of size 1 x 2 cm were made with new grease-free slides and allowed to air dry. The air dried smears were then fixed by gently passing over a flame 2–3 times. The smears including positive and negative controls were stained with Ziehl-Neelsen (ZN) technique. Examination and reporting of stained slides were performed by light microscopy (22).

**Culture and identification**: Portion of each specimen sediment simultaneously inoculated into solid Lowenstein Jensen (2–3 drops) and BACTEC MGIT 960 media (Becton Dickinson, Franklin Lakes, NJ07417, USA) (0.5 ml). LJ medium cultures incubated in a 37°C for a maximum of eight weeks (monitored every week) whereas liquid culture was incubated in an automated BACTEC MGIT 960• machine (Becton Dickinson Diagnostic Instrument Systems) for a maximum of 42 days to report negative. Cultures exhibiting growth were subjected to light microscopy for the presence of acid-fast bacteria and inoculated to blood agar (BAP) before being considered as positive for *Mycobacteria* (22).

Isolates grown on blood agar after overnight incubation and no acid fast bacilli (AFB) seen on Ziehl-Neelson staining were considered as contaminants. However, isolates which showed no growth on BAP and positive for AFB were considered as *Mycobacteria*. In addition, isolates grown on BAP and AFB seen on Ziehl-Neelsen staining were considered as the presence of both contaminants and *Mycobacteria* (22).

**Capilia TB-Neo rapid test**: Capilia TB-Neo rapid test was used to classify the mycobacteria into *Mycobacterium tuberculosis* complex (MTBC) and probable Nontuberculous mycobacteria. The Capilia TB-Neo Rapid test negative and positive growth were considered as probable NTM and MTBC respectively. Confirmation and characterization of the probable NTM was performed by Hain's GenoType® Mycobacterium CM and GenoType® Mycobacterium AS molecular genetic assays.

**DNA extraction and GenoType Mycobacterium CM/AS assay**: Nontuberculous DNA was extracted from heat-inactivated AFB isolates. Briefly, bacteria suspended in 500 µl sterile water or 1 ml directly from positive liquid media were inactivated at 80 °C for 20 min, then ultrasonicated at 35 kHz and heated at 100 °C for 10 min each treatment and centrifuged at 16,100 g two times for 5 minutes. The supernatant was taken as template DNA (23).

GenoType® Mycobacterium CM/AS (HainLifescience GmbH, Nehren, Germany) assays were performed using the template DNA according to the manufacturer's instructions. The GenoType CM assay was used to identify the common NTM species. GenoType AS assay was used to identify additional NTM species when the species are undetermined by the former assay. Those tests are based on the DNA strip technology. Three controls (conjugate, universal, and genus) are included in each strip (23).

**Drug susceptibility test**: Phenotypic method using BACTEC-MGIT 960 SIRE Kits (Franklin Lakes, NJ, USA) was done on pure isolates of MTBC. It was performed for the first line antituberculosis drugs using BACTEC MGIT 960 media. The critical concentration of each drug was 1µg/ml, 0.1µg/ml, 1µg/ml, 5µg/ml and 100µg/ml for streptomycin, isoniazid, rifampicin, ethambutol and pyrazinamide respectively. The MGIT 960 system automatically interpreted the results as susceptible or resistant (22).

**Statistical analysis**: Data were collected, entered, and analyzed using SPSS version 20 software. Categorical variables were described as proportion. Binary logistic regression was performed to check the presence of an association between MTBC and independent variables. A p-value less than 0.05 was considered as statistically significant.

**Ethics approval and consent to participate**: Ethical clearance was obtained from the Ethical Review Committee of Medical Laboratory Sciences, College of Health Sciences, Addis Ababa University. Written informed consent was also obtained from all eligible study participants or assent from the guardians of participants younger than 18 years. Laboratory confirmed cases were managed by the clinicians.

## Results

**Characteristics of study participants**: A total of 275 presumptive pulmonary tuberculosis patients with a predominance of males (150, 54.5%) were enrolled. The mean age was 43 years (± 6.6 SD) and the majority (33.1%) fell in the age range of 31–45 years (Table 1).

**Table 1:** Demographic, health and behavioral characteristics of 275 presumptive tuberculosis patients at St. Paul's Hospital Millennium Medical College, 2016

Variable	Frequency	Percent
**Sex**		
Male	150	54.5
Female	125	45.5
**Residence**		
Rural	95	34.5
Urban	180	65.5
**Age**		
16–30	78	28.4
31–45	91	33.1
46–60	62	22.5
≥ 61	44	16
**Educational level**		
Illiterate	102	37.1
< 6	42	15.3
6–12	105	38.2
Higher education	26	9.5
**Occupation**		
Farming	84	30.5
Office work/student	69	25.1
House wife	75	27.3
Others	47	17.1
**Previous TB treatment**		
Yes	75	27.3
No	200	72.7
**HIV status**		
Reactive	36	13.1
Non-reactive	77	28
Unknown	162	58.9
**Diabetes mellitus**		
Yes	19	6.9
No	54	19.6
Unknown	202	73.5
**Smoke cigarette**		
Yes	16	5.8
No	259	94.2
**Drink alcohol**		
Yes	58	21.1
No	217	78.9
**Chew Kchat**		
Yes	21	7.6
No	254	92.4

Most (96.4%) of the participants were ambulatory patients. About 36(13.1%), 75(27.3%) and 19(6.9%) of the study participants had HIV infection, history of previous tuberculosis treatment and diabetes mellitus respectively (Table 1).

**Clinical features and chest x-ray results of study participants**: Persistent cough for more than two weeks and chest pain were observed in 259(94.2%) and 228(82.9%) participants respectively. Of 224 participants with known chest x-ray results, 111(49.6%) showed different types of abnormalities (Table 2).

**Table 2:** Distribution of clinical and chest x-ray results of 275 presumptive tuberculosis patients at St. Paulo's hospital millennium medical college, 2016

Variable	Frequency	Percent
**Cough ≥ 2 weeks**		
Yes	259	94.2
No	16	5.8
**Hemoptysis**		
Yes	77	28
No	198	72
**Night sweeting**		
Yes	151	54.9
No	124	45.1
**Fever**		
Yes	182	66.2
No	93	33.8
**Chest pain**		
Yes	228	82.9
No	47	17.1
**Chest x-ray result**		
Normal	113	41.1
Abnormal	111	40.4
Unknown	51	18.5

**Magnitude of *mycobacterium tuberculosis* complex and nontuberculous mycobacteria**: Twenty-nine (10.6%, 29/275) morning sputum samples were found to be culture positive for *Mycobacteria*. Of the 29 participants with confirmed mycobacteria, 26(89.7%) were MTBC and the remaining 3(10.3%) were NTM. Sputum smear microscopy identified TB in 12 (45.2%) cases. These resulted in an overall magnitude of 9.5% (26/275) for MTBC and 1.1% (3/275) for NTM. There was no coisolation of MTBC and NTM in the sputum samples examined.

Of the three isolated nontuberculous mycobacteria, two were unidentified NTM and one was identified as *Mycobacterium peregrinum*. Two of the isolated NTM were rapid growers. All the NTM were negative for direct sputum smear microscopy.

***Mycobacterium tuberculosis* magnitude and chest x-ray results**: We assessed the chest x-ray profiles of 224 study subjects out of 275 patients. The chest x-ray results of 51 patients (18.5 %, 51/275) were not available during data mining from the patient card. The radiologist reported normal chest x-ray results for 113 subjects (113/ 224). However, 8 patients (7.1%, 8/113) were positive for MTBC using culture (Table 3).

**Table 3:** Frequency of *Mycobacterium tuberculosis* complex isolates along with chest x-ray results at St. Paul's Hospital Millennium Medical College, 2016

Chest x-ray result	Total	MTBC Isolate Number(%)	Smear positive	Smear negative/ culture positive
Normal	113	8(32%)	2	6
Abnormal	111	17(68%)	10	7
Total	224	25(100)	12	13

**MTBC**- *Mycobacterium tuberculosis* complex

Regarding nontuberculous mycobacteria, two were from patients who had normal chest xray results and one from a patient who had abnormal chest x-ray result.

**Drug resistant profile of *Mycobacterium tuberculosis* complex isolates**: Of the twenty six isolated *Mycobacterium tuberculosis* complex, 6(23.1%) isolates were resistant to at least one drug. The highest proportions of any drug resistance were observed in Streptomycin 4(15.4 %) followed by Isoniazid 3(11.5 %). Two MDR cases (7.7%) were isolated from patients who had a previous tuberculosis treatment history (Table 4).

**Table 4:** Distribution of drug resistance pattern of 26 isolated *Mycobacterium tuberculosis* from patients at St. Paulo's hospital millennium medical college, 2016

Sensitivity/Resistance	Number (%)
Sensitive to all drugs	20(76.9%)
Single resistance	
Streptomycin	3(11.5%)
Isoniazid	1(3.8%)
Double resistance	
Rifampicin and Isoniazid	1(3.8 %)
Triple resistance	
Rifampicin, Isoniazid, Streptomycin	1(3.8%)

***Mycobacterium tuberculosis* complex (MTBC) and associated factors**: Larger proportion of females (9.6%, p=0.940), participants within the age range of 16–30 years (14.1%, p=0.119) and HIV infected patients (8.3%, p=0.723) were TB positive. The pulmonary TB positive and negative individuals had no significant differences concerning educational status, occupation, residence, marital status, HIV status, DM status, alcohol consumption, and smoking cigarette (Table 5).

**Table 5:** Association between socio-demographic, behavioral, clinical characteristics and MTBC positivity of study participants at St. Paul's Hospital Millennium Medical College, 2016

Variable	Positive N (%)	COR (CI; 95%)	P-value	
**Sex**				
Male	14(53.8)	1.119(0.443–2.824)	0.940	
Female	12(46.2)	**I**		
**Residence**				V0.393
Rural	19(73.1)	1.484(0.6–3.666)	0.393	
Urban	7(26.9)	**I**		
**Age**				
16–30	11(42.3)	0.290 (0.061–1.374)	0.119	
31–45	9(34.6)	0.434(0.090–2.099)	0.299	
46–60	4(15.4)	0.690(0.121–3.947)	0.677	
≥ 61	2(7.7)	**I**		0.984
**Educational level**				
Illiterate	5(19.2)	2.53(0.564–11.36)	0.226	
< 6	5(19.2)	0.965(0.210–4.427)	0.964	
6–12	13(50)	0.923(0.243–3.511)	0.907	0.9070.907
Higher education	3(10.3)	**I**		
**Occupation**				
Farming	8(30.8)	0.884(0.251–3.107)	0.847	
Office work/student	7(26.9)	0.904(0.259–3.271)	0.904	
House wife	7(26.9)	0.824(0.227–2.989)	0.824	
Others	4(11.515.4)	**I**		
**Previous TB treatment**				00
Yes	5(19.2)	1.642(0.596–4.526)	0.337	
No	21(80.8)	**I**		
**HIV status**				
Reactive	3(11.5 )	0.764(0.172–3.388)	0.723	
Non-reactive	5(17.2)	**I**		
**Diabetes mellitus**				
Yes	2(7.7)	1.70 (0.333–8.682)	0.521	
No	9(34.6)	**I**		
**Smoke cigarette**				
Yes	2(7.7)	0.715(0.153–3.335)	0.669	
No	24(92.3)	**I**		
**Drink alcohol**				
Yes	7(24.1)	0.699(0.279–1.754)	0.446	
No	19(75.9)	**I**		

**I-**Reference category, **COR-** Crude Odds Ratio, **CI-**Confidence interval, **N-**Number, **TB-**Tuberculosis

## Discussion

Isolation and detection of mycobacteria play an important role in the control and prevention of tuberculosis and tuberculosis like diseases (24). During the four month period of our study, a total of 275 presumptive pulmonary TB patients were enrolled, and 29(10.6%) of them were positive for *Mycobacteria* infection. Of these, the majority were MTBC 26(89.7%). *M.peregrinum* was the NTM species identified.

A similar study on identification of mycobacteria in Nigeria reported that out of 270 suspected pulmonary tuberculosis patients, 26(9.5%) were infected with mycobacteria (25), which is comparable to our finding. However, higher isolation rates were reported from Tanzania, (32.5%) (26) and Ethiopia, (17.4%) (15).

The overall magnitude of bacteriologically confirmed pulmonary tuberculosis in our study (9.5%) was lower than previous reports in Ethiopia (16.5%) (16), Tanzania (22.8%) (26) and Nigeria (28%) (27). However, our finding showed a higher burden than another study conducted in Nigeria (6.3%) (25). These may reflect the variations in the study population and laboratory method. Of the cultured confirmed TB, 32% (8/25) patients had normal chest x-ray results. This implies that the use of sputum culture in the diagnosis of active tuberculosis improves detection rates and ultimately leads to improved patient outcomes and reduced transmission of *M. tuberculosis*.

Previous reports in Ethiopia from National Tuberculosis Reference Laboratory (6%) and Saint Peter Tuberculosis Specialized Hospital Laboratory (7%) indicated presence of NTM without confirmation and speciation (15,20). The present study unlike the former revealed higher percentage (10.3%) of confirmed nontuberculous mycobacteria. This finding is consistent with studies done in Iran (10.2%) (3) and Tanzania (9.7%) (26), but higher isolation rate was observed in USA (14%) (14) and Nigeria (15%) (27). This might be due to the variation in study population and laboratory method.

The distribution of NTM species significantly varies across regions (10). Of the 3 NTM isolated in this study, molecular technique showed that one NTM was typed as *M. peregrinum*. Studies carried out in some African countries identified *M. peregrinum* and other NTM species from sputum samples of presumptive pulmonary tuberculosis patients (28,29,8). Additionally, *M. peregrinum* implicated as a possible causative agent of pulmonary disease in human (30,31). However, the NTM isolates in this study were not evaluated by the diagnostic criteria for NTM disease published by the American Thoracic Society (ATS).

In this study, a resistance proportion to at least one of the five anti-tuberculosis drug tested was 23.1% which is comparable to reports in Addis Ababa, Ethiopia (22.3%) (17) and Libya (24.1%) (32). However, higher resistance proportion was reported in other studies done in Ethiopia (27.4%) (18) and Kenya (30.1%) (33). This variation could be due to the fact that the latter two studies were conducted at referral sites for TB cases. A study done in East Gojam, Ethiopia, reported slightly lower level (20.23%) of drug resistance than our study (19).

This study has some limitations. There was no follow-up of patients who were positive for nontuberculous mycobacteria even though reported to the concerned physician. The study was also limited to patients who were attended to in one hospital, but the information reported in this study could be a significant contribution to the existing knowledge on the burden of MTBC and nontuberculous mycobacteria.

In conclusion, this study depicted that the majority of the isolated *Mycobacteria* from sputum samples of presumptive pulmonary tuberculosis patients were *Mycobacterium tuberculosis* complex (89.7%) and low extent of nontuberculous mycobacteria (10.3%). Of the isolated MTBC, 76.9% were susceptible to five anti-tuberculosis drugs. Therefore, further large scale study is needed to substantiate those results and to consider NTM as a differential diagnosis of pulmonary tuberculosis like disease.
